# Has government aging governance alleviated rural multidimensional relative poverty? Evidence from the China Rural Revitalization Survey

**DOI:** 10.3389/fpubh.2025.1524879

**Published:** 2025-05-01

**Authors:** Heng Wang, Shu Yang, Xianfeng Zeng, Yu Wang

**Affiliations:** ^1^School of Economics and Finance, Xi’an International Studies University, Xi’an, Shaanxi, China; ^2^Global South Economic and Trade Cooperation Research Center, Xi’an, Shaanxi, China; ^3^Rural Development Institute, Chinese Academy of Social Sciences, Beijing, China

**Keywords:** population aging, government governance, rural households, multidimensional relative poverty, China Rural Revitalization Survey

## Abstract

**Background:**

Population aging poses a significant global challenge, particularly in rural areas where it increases economic burdens and living pressures, potentially pushing families into poverty.

**Methods:**

This study utilizes data from the 2020 and 2022 China Rural Revitalization Survey (CRRS) and applies the Alkire-Foster (A-F) method to measure rural multidimensional relative poverty. A fixed effects model is used to systematically examine the mechanisms and impacts of government aging governance on rural multidimensional relative poverty.

**Results:**

The analysis yields three key findings: (1) Government efforts in aging governance have played a significant role in alleviating rural multidimensional relative poverty, through improvements in grassroots democratic quality and network conditions. (2) Rural households in China’s eastern regions benefit more from aging governance due to more developed infrastructure and broader social services. (3) The impact of aging governance on reducing multidimensional relative poverty is more pronounced in younger households compared to older adult households.

**Conclusion:**

This study underscores the role of government aging governance in addressing rural poverty and provides policy recommendations to develop a localized multidimensional relative poverty assessment system, improve governance capacity in aging-related affairs, and enhance grassroots democratic quality and rural digital infrastructure.

## 1 Introduction

Global population aging has become an irreversible trend. According to the World Health Organization, by 2030, individuals aged 60 and above will constitute one-sixth of the global population; by 2050, the number of people aged 60 and older will have doubled, reaching 2.1 billion (1). The rapidly increasing older adult population places significant pressure on governments worldwide, particularly in terms of pension systems, social security, and healthcare services. Enhancing the quality of social protection and caregiving services for aging populations has thus become an urgent issue that nations around the world must address.

China, as a populous nation, has seen a rapid rise in its older adult population, with the proportion of those aged 65 and above increasing from 6.96% in 2000 to 15.4% in 2023 (2). On one hand, due to large-scale labor migration, rural areas are experiencing faster and more severe population aging. Data shows that in 2000, the aging rates in rural and urban areas were 10.9 and 9.7%, respectively, but by 2020, these figures had risen to 23.8 and 15.8%, respectively (3). On the other hand, rural China faces greater challenges in responding to population aging due to the uneven distribution of older adult care resources. For a long time, rural older adult people have been disadvantaged compared to their urban counterparts in terms of health, medical care, and social security. In terms of health, the self-reported health rate of rural older adult was 48.22, 11.3% lower than in urban areas (4). In terms of medical care, rural areas face weak medical infrastructure and the outflow of healthcare personnel. In terms of social security, the average pension received by rural residents in 2022 was RMB 204.7 per month, accounting for only 35.2% of the average minimum living standard in rural areas (5). Overall, existing research suggests that the poverty rate among the older adult in rural areas is higher than the overall poverty rate in rural regions (6).

To enhance the multidimensional well-being of the rural older adult population, the Chinese government has implemented a series of policy measures. These include the introduction of the New Rural Social Pension Insurance system in 2009. The State Council of the People’s Republic of China (SCPRC), in its 2013 document Several Opinions on Accelerating the Development of the Older adult Care Service Industry, emphasized increasing investment in grassroots and rural older adult care services to strengthen pension security in rural areas. The launch of pilot reforms in 2016 aimed at establishing a multi-tiered older adult care service system based on home care, supported by communities, and supplemented by institutions. The 2022 *Notice on Issuing the* “*14th Five-Year Plan” for National Aging Development and the Older adult Care Service System* aims to establish a rural mutual-aid older adult care network. The goal of these initiatives is to genuinely improve the welfare of the rural older adult population and promote comprehensive economic and social development in rural areas in the context of an aging society.

Government governance plays a crucial role in rural poverty alleviation (7). Effective governance, through the development of sustainable pension systems, the formulation of social security policies, the improvement of rural infrastructure, and the provision of health and social services, can significantly reduce the living pressures of the rural older adult population, improving their quality of life and thereby alleviating rural multidimensional relative poverty. For example, the Japanese government has promoted rural revitalization by strengthening social security and developing agricultural cooperatives. Its social security system, which includes social insurance, public assistance, and social welfare, has enhanced rural older adult people’s ability to cope with risks and ensured their quality of life. Conversely, the absence of effective governance can exacerbate poverty in rural areas, placing greater demands on the government’s social governance capacity (8). In Brazil, the assistance-based rural pension system has played a significant role in addressing the challenges posed by aging, narrowing the urban–rural wealth gap, and ensuring the livelihood of the rural older adult, though it has also placed substantial financial pressure on the government. Therefore, effective aging governance is key to mitigating the potential adverse effects of population aging. For China, addressing the challenges of rural aging under conditions of unequal urban–rural resource distribution requires sound government governance to allocate resources across regions, ensuring that the rural older adult have access to adequate living conditions and healthcare.

Based on the above, this paper takes China as a case study to explore whether government aging governance can alleviate poverty and enhance the multidimensional well-being of the rural older adult in the context of population aging. This topic not only deepens our understanding of government governance but also offers innovative insights into the global challenges of aging governance. Against this backdrop, the paper focuses on the well-being of the rural older adult, utilizing data from the 2020 and 2022 China Rural Revitalization Survey (CRRS), as well as government work reports and statistical yearbooks, to systematically measure the impact and mechanisms of government aging governance on rural older adult well-being. Compared to existing research, this paper offers several potential contributions. First, it measures the level of government aging governance to assess its impact on the well-being of the rural older adult. Second, it analyzes the mechanisms through which government aging governance affects rural multidimensional relative poverty, addressing the limitations of current research, which is largely confined to theoretical and macro-level data analysis. The structure of this paper is as follows. Section 1 introduces the research topic. Section 2 reviews the relevant literature. Section 3 describes the empirical modeling and data sources. Section 4 presents the empirical results and analysis. Section 5 explores heterogeneity and mechanism analyses. Finally, Section 6 concludes the paper with policy recommendations.

## 2 Literature review and research hypotheses

### 2.1 Literature review

#### 2.1.1 Research on rural multidimensional relative poverty

The academic understanding of poverty has evolved from a focus on income poverty to multidimensional poverty and, eventually, to relative poverty, with research becoming more comprehensive and in-depth. Traditional absolute poverty is primarily based on minimum standards of income and physiological needs, while multidimensional relative poverty focuses on an individual’s relative position in society and their opportunities for development. Shorrocks (9) introduced the concept of relative poverty, emphasizing that poverty is not only about the lack of resources but also about social exclusion and the inability to participate in societal activities. Sen (10) proposed the concept of multidimensional poverty, highlighting deprivations in areas such as health, education, and living conditions. As research has progressed, scholars have refined the measurement of multidimensional poverty. Decancq, Fleurbaey and Maniquet (11) developed a weighting scheme for multidimensional relative poverty based on individual preferences. Alkire, Nogales (12) established a dynamic multidimensional poverty model based on the Alkire-Foster (A-F) method. Kumar, Chakraverty and Sethi (13) used principal component analysis to construct a composite multidimensional poverty index. Ogwang and Lamarche (14) proposed a hybrid Watts-MPI approach for measuring multidimensional poverty, which addresses some of the information loss present in conventional multidimensional poverty measures.

In recent years, scholars have increasingly applied the concept of multidimensional relative poverty to the study of rural relative poverty and poverty governance in China. The use of multidimensional relative poverty allows for more effective identification of transitional and potential poverty groups, enables the evaluation of the effectiveness of China’s multidimensional poverty alleviation efforts, and aids in the formulation of more targeted policies to alleviate relative poverty and prevent poverty recurrence. Existing studies primarily assess relative poverty using dimensions such as income level, health and education, living conditions, employability, and personal perceptions (15–18), providing valuable references for identifying multidimensional poverty in rural China. Some scholars focus on the poverty conditions of specific groups. Zeng, Zhao (19) constructed a multidimensional relative poverty index for the rural older adult, incorporating health, social, psychological, and material dimensions. Peng (20) measured and compared the relative poverty of rural women in China across six dimensions: economy, health, culture, spiritual life, social relationships, and rights.

#### 2.1.2 The impact of population aging on rural multidimensional relative poverty

The growth of population aging has negative implications for poverty (21). As China enters an aging society, the aging process exacerbates rural labor shortages and increases the burden of social care and economic support (22), leading to a rising trend of multidimensional relative poverty among the rural older adult (19). Population aging has thus become a key factor influencing rural multidimensional relative poverty. Current research on the impact of population aging on multidimensional relative poverty primarily explores the incidence and effects of multidimensional and relative poverty in the context of aging. In the study of multidimensional poverty, Zeng, Zhao (19) used the fuzzy set approach to calculate a multidimensional relative poverty index for the older adult and found that health is the dominant factor in older adult multidimensional relative poverty. Hou, Zhou and Jiang (23) analyzed the relationship between multidimensional energy poverty and depression levels among the older adult. Chou, Puthenparambil (24) compared differences in the rates of older adult care poverty between East Asia and Northern Europe. In the study of relative poverty, scholars generally use relative income as a proxy for relative poverty. Cui and Chang (25) found that relative income has a significant negative impact on older adult health outcomes, while Tanaka, Miyawaki (26) identified relative poverty as a major risk factor for mortality among retired men. Furthermore, Cui and Chang (25) found that the migration of children can reduce the risk of rural older adult falling into relative poverty.

#### 2.1.3 The impact of government aging governance on rural multidimensional relative poverty

Government governance plays a crucial role in reducing rural poverty and inequality (27). Through mechanisms such as taxation, social security, and transfer payments, government governance can strengthen redistributive systems and improve the social security conditions of the older adult, effectively alleviating poverty levels among the older adult population (28). Moreover, the effectiveness of government governance in reducing rural older adult poverty lies in its ability to mobilize resources and prioritize social assistance for the older adult, sick, disabled, and others who cannot maintain basic living standards (29). Existing literature mainly explores how government policies and spending impact rural older adult poverty. Anderson, d’Orey (30) using meta-regression analysis, found that government expenditure effectively reduces income poverty in low-and middle-income countries. Wen and Sun (31) through a PSM-DID method, evaluated the impact of China’s New Rural Pension Scheme on alleviating relative poverty among rural households. Ajefu and Ogebe (32) found that increased intergovernmental transfers led to a decrease in the multidimensional poverty index of households. Yi, Huo (33) using data from Chinese households, confirmed that optimized governance of new rural collective economic organizations reduces relative poverty in rural areas. The study by Hoang-Duc, Nguyen-Thu (34) demonstrated that government support contributes to reducing multidimensional poverty levels among impoverished populations.

The existing literature on population aging, government governance, and rural multidimensional relative poverty is abundant, providing a solid theoretical foundation for this study. However, most research has been conducted at the theoretical and macro levels, with relatively few micro-level data studies, resulting in a lack of specificity. This paper connects government aging governance with rural multidimensional relative poverty and validates the effectiveness of aging governance in alleviating rural multidimensional relative poverty. The findings offer empirical evidence and significant practical implications for policymaking.

### 2.2 Research hypotheses

Government aging governance can effectively alleviate rural multidimensional relative poverty by promoting the welfare of older adults and reducing the burden on young adults. From the perspective of the older adult group, government aging governance promotes the quality of life, happiness and social participation of the rural older adult by improving the old-age security system, improving the medical and health service system for the older adult, and providing re-employment positions for the older adult (35), directly alleviating the multidimensional relative poverty of the rural older adult group. From the perspective of young adults (we consider young adults here as laborers who have not yet reached the age of retirement, as opposed to the older adult), the government’s increased investment in old-age pension and medical care, and the promotion of the construction of medical care facilities for the older adult, indirectly creates jobs related to nursing and medical care, and provides employment opportunities for young adults in rural areas, which in turn raises their income levels and social participation, and alleviates their multidimensional relative poverty. Meanwhile, in the traditional Chinese concept, children have the obligation to support the older adult, a concept that is even more pronounced in rural China (36). The alleviation of the multidimensional poverty level of the older adult actually also reduces the burden of the young people’s old-age pension. With the government’s support for the older adult, young adults in rural families will spend less time and capital on caring for the older adult, and will be able to invest more time and capital in production, thus raising the family’s income level and quality of life, and alleviating the multidimensional relative poverty level of rural families. In summary, government governance of aging actually enhances the ability of rural households as a whole to withstand the risk of poverty. Therefore, we propose the following research hypotheses:

*H1*: Government governance of aging can significantly alleviate the level of multidimensional relative poverty in rural areas.

The effective implementation of the governmental governance of aging policy cannot be separated from the improvement of the quality of grassroots democracy. As grassroots self-governance organizations, village committees play a crucial role in promoting the governance of aging policy. In the past grassroots governance process, the older adult belonged to a minority and vulnerable group, coupled with the generally low level of social recognition of the value of the older adult, although most of the older adult have the intention to participate in community governance, but the actual degree of participation is very low (37), which makes the older adult often neglected in community governance. Under the framework of aging governance, the government encourages the participation of the older adult in village affairs and election activities, which makes the subjects of participation in basic democracy more complete, and improves the participation and voice of the older adult in rural governance, so that their demands are more valued in decision-making. As the main implementers of the government’s policies on aging governance, village committees are responsible for promoting pension insurance coverage, expanding non-agricultural employment, and promoting the construction of a pension security system. Generally speaking, where the level of government governance is higher, higher-level governments usually allocate more special funds to grass-roots self-governance organizations, which village committees use to expand the supply of old-age services and improve the infrastructure of activity centers and nursing homes for the older adult, thereby raising the level of welfare. The improvement in the quality of grassroots democracy enables older groups to monitor policy implementation more effectively, ensuring that special funds and old-age services truly benefit the rural older adult, thereby alleviating their multidimensional relative poverty. Therefore, we propose the following research hypothesis:

*H2*: Government governance of aging alleviates multidimensional relative poverty in rural areas by improving quality of grassroots democracy.

With the development of the digital economy, older people face significant challenges related to the digital divide. In the sample surveyed for this study, 71.91% of rural older adult households reported difficulties in using 4G/5G mobile phones, and due to barriers to internet use, the older adult have difficulty accessing resources such as online healthcare and distance education, exacerbating poverty in the health and education dimensions. To address this challenge, in 2020, the General Office of the State Council and the Ministry of Industry and Information Technology issued the Implementation Plan for Effectively Resolving Difficulties in the Use of Smart Technologies for the Older adult and the Special Action Plan for the Transformation of Internet Applications for Older adult and Easily Accessible to the Older adult, which focuses on the construction of an age-adapted digital society. The government’s construction of an age-friendly digital society is an important manifestation of the central government’s active response to population aging and the national strategy of network power under the national strategy, but also the reason why the government’s aging governance is able to improve network conditions. As for the older adult, the improvement of network conditions, such as equipping smartphones and providing Internet access venues, has enabled them to gain more opportunities for information access and social participation. By providing better network conditions, older people can have easier access to healthcare resources, educational resources and social services, thus improving their health and knowledge (38) and reduce poverty caused by illness and lack of information. In addition, good network conditions promote the development of rural e-commerce, provide re-employment opportunities for the older adult, and increase the non-farm income sources of rural residents, thereby also promoting their social participation and income levels. Therefore, we propose the following research hypothesis:

*H3*: Government governance of aging alleviates multidimensional relative poverty in rural areas by improving network conditions.

## 3 Empirical modeling and data sources

### 3.1 Model specification

The econometric model constructed in this paper is as follows:


(1) $$MP_{\mathrm{it}}=\alpha +\beta OG_{\mathrm{it}}+\lambda X_{\mathrm{it}}+\varphi_{i}+\gamma_{j}+\varepsilon_{\mathrm{it}}$$

In Equation 1, the dependent variable $MP_{\mathrm{it}}$ represents whether a household is in a state of multidimensional relative poverty. The key explanatory variable $OG_{\mathrm{it}}$ represents the level of government aging governance in prefecture−level city i in year t . $X_{\mathrm{it}}$ denotes control variables. Additionally, prefecture-level fixed effects $\varphi_{i}$ , time fixed effects $\gamma_{j}$ , $\varepsilon_{\mathrm{it}}$ is the random error term, clustered at the household level.

### 3.2 Variable measurement

#### 3.2.1 Dependent variable

This study draws on the work of current scholars regarding the construction and measurement of the multidimensional relative poverty index system for households (39–43). Building on existing research, we employ the Alkire-Foster (AF) method, developed by the Oxford Poverty and Human Development Initiative (OPHI), to identify and measure the multidimensional relative poverty of households. Five dimensions are selected to identify multidimensional relative poverty: educational attainment, health status, subjective well-being, social participation, and income status (44, 45). The indicators are shown in Table 1. For 0–1 variables, a threshold of 1 is applied, meaning that a household is considered relatively poor in that dimension if the value is 1. For continuous variables, following previous studies, the threshold is set at 80% of the variable’s median value. If the household’s value falls below this threshold, it is considered relatively poor in that dimension.

(1) Identification of unit-level relative poverty. Suppose the sample size of households is i and and the number of multidimensional poverty indicators is j . Construct an i×j matrix Y , where the non−negative elements $y_{ij}$ in matrix Y represent the value of the j -th indicator for the i -th household. On this basis, construct a matrix $Z=\left(z_{1},z_{2},\cdots ,z_{m}\right)$ , a vector composed of deprivation thresholds for the corresponding indicators, where $z_{j}$ is the deprivation threshold for indicator j . When $y_{ij}\leq z_{ij}$ , the household is in a state of relative poverty for indicator j and is assigned a value of 1; otherwise, the household is not considered deprived in that dimension and is assigned a value of 0. The function is constructed as shown in Equation 2:

(2) $$\lambda =\{\begin{array}{c} 1,y_{ij}\leq z_{j}\\ 0,\mathrm{otherwise} \end{array}$$

(2) Multidimensional poverty relative identification. Assign a weight $w_{j}$ to each indicator for the samples. Let $G_{i}\left(k\right)$ represent the weighted total deprivation score for the i -th household, as shown in Equation 3:

(3) $$G_{i}\left(k\right)=\overset{m}{\underset{j=1}{\sum }}w_{i}\cdot \lambda_{ij}$$

**Table 1:** Selection and description of multidimensional relative poverty indicators.

Dimension	Variable	Description	Threshold	Weight
Education	Education	Average years of education of household members	Median * 0.8	1/5
Life quality	Drinking water	Whether the household uses tap water	1 if not using tap water, otherwise 0	1/25
	Living environment	Whether the household has a sanitary toilet	1 if not using a sanitary toilet, otherwise 0	1/25
		Whether the village has unified garbage disposal	1 if no unified disposal, otherwise 0	1/25
	Health status	Health Status Compared to Peers	Median * 0.8	1/25
		Body Mass Index (BMI)	Adults <18.5 or Children <16.5	1/25
Subjective well-being	Life satisfaction	Household head’s satisfaction with last year’s household income	Median * 0.8	1/25
		Household head’s satisfaction with current housing situation	Median * 0.8	1/25
		Household head’s satisfaction with your home’s living environment	Median * 0.8	1/25
		Household head’s satisfaction with local public security	Median * 0.8	1/25
	Future expectations	How householders feel their lives have changed in 5 years time	Median * 0.8	1/25
Social participation	Employment status	Current employment status	1 for not employed, 0 otherwise	1/15
	Social security	Whether to participate in urban and rural residents’ medical insurance	Not participating is 1, otherwise 0	1/15
	Old age security	Whether to participate in pension insurance	Not participating is 1, otherwise 0	1/15
Income status	Income level	Total annual income	Median * 0.8	1/10
	Change in income	Change in head of household’s estimated income for the year over the previous year	Median * 0.8	1/10

Here k represents the deprivation threshold, and $k\in \left[0,1\right]$ , The larger the value of $G_{i}\left(k\right)$ the deeper the level of multidimensional poverty for the household. When $G_{i}\left(k\right)\geq k$ , the household is in a state of multidimensional relative poverty. Following the method of Alkire and Foster (39), this paper applies equal weights to each indicator j .

(3) Calculation of the Multidimensional Relative Poverty Index. The multidimensional relative poverty index is calculated using the following Equations 4–6:

(4) $$H=\frac{q}{n}$$

(5) $$A=\frac{1}{q}\overset{n}{\underset{i=1}{\sum }}G_{i}\left(k\right)$$

(6) MPI=H⋅A

Where *H* represents the incidence of relative multidimensional poverty, *A* represents the average intensity of deprivation in multidimensional relative poverty, and *MPI* is the multidimensional relative poverty index for households.

**Figure 1:**
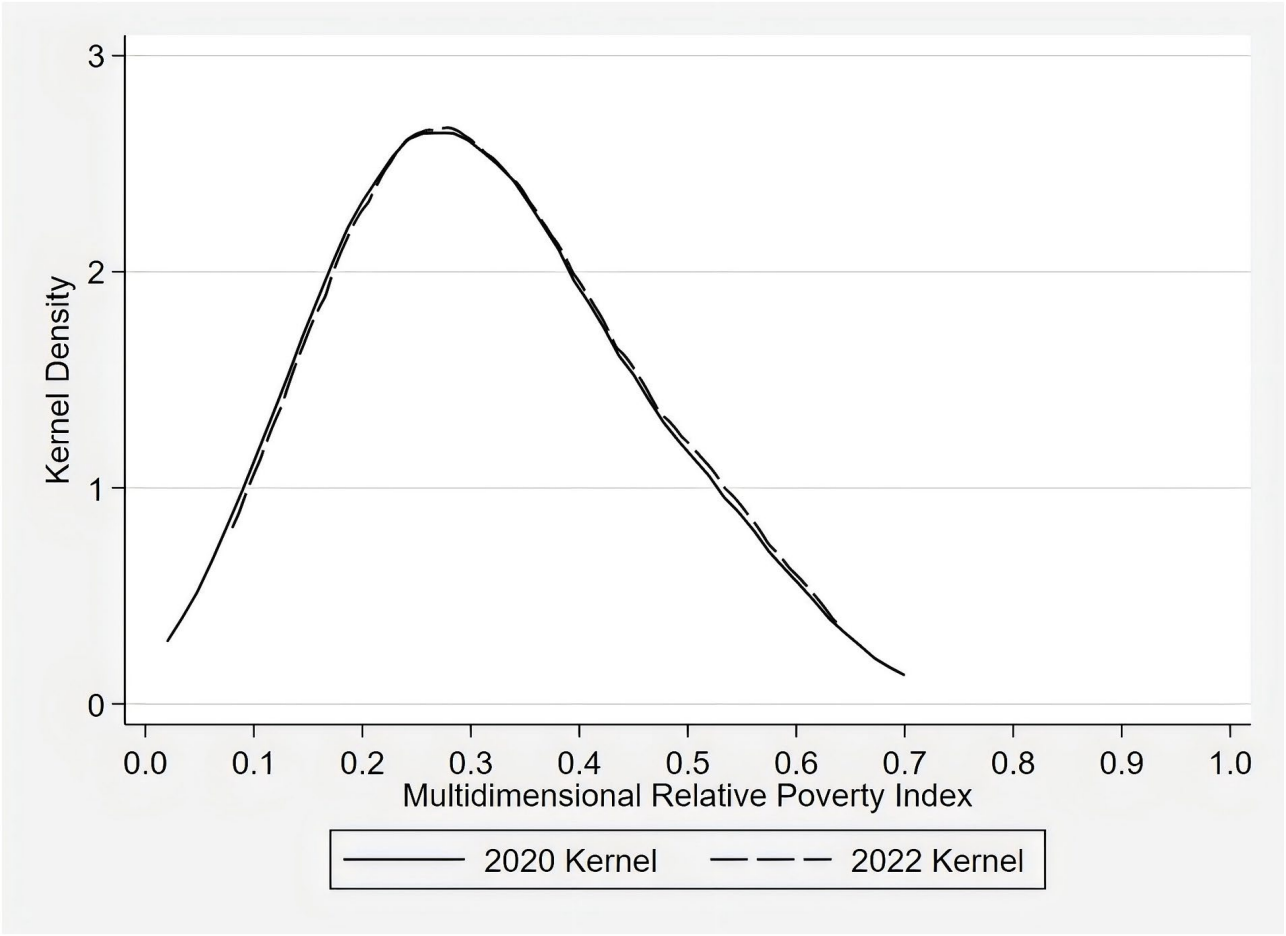
Kernel density curve of multidimensional relative poverty of rural households.

Based on the above steps for measuring rural multidimensional relative poverty, the multidimensional relative poverty index for households was obtained. To visually display the clustering degree of different household groups under varying levels of multidimensional relative poverty, a Kernel Density function was further applied to describe the multidimensional relative poverty scores of households in 2020 and 2022, with the results shown in Figure 1. Overall, there is little change in the distribution of rural household poverty in the 2 years. The kernel density curve peaks on the left, with a relatively broad peak and a “right tail” pattern, indicating that while the overall level of poverty among households is relatively low, there are still some families experiencing higher levels of poverty.

Using the above calculations, we construct the explanatory variable multidimensional relative poverty (MP) for this paper. A binary variable is used, with *k* = 0.3 as the threshold for rural multidimensional relative poverty. If k≥0.3 , the household is considered to be in a state of multidimensional relative poverty, and *MP* is assigned a value of 1. If k<0.3 , the household is not considered to be in a state of multidimensional relative poverty, and *MP* is assigned a value of 0.

#### 3.2.2 Key explanatory variable

The integration of the Party and government is a prominent feature of China’s administrative system. Essentially, the relationship between the Communist Party of China (CPC) and the Chinese government is one of principal-agent. The Party acts as the principal, providing overall guidelines, while state bureaucratic institutions are responsible for formulating and implementing specific policies and reporting back to the Party (46). Government work reports serve as a key medium for the government to report on its performance, showcase its policy initiatives, and outline strategic plans. Therefore, these reports fully reflect the government’s attention and governance efforts across various sectors of economic, cultural, and social development (47, 48). Based on this, the present study selected 101 aging governance-related terms from reports by the National Development and Reform Commission of China and other information related to the older adult care industry. The frequency of these aging governance terms in government work reports was counted, and the proportion of these terms relative to the total length of the text was used to measure the key explanatory variable governmental aging governance level (OG). The categories of terms and specific keywords are shown in Table 2. As an important administrative level within China’s governance system, prefecture-level governments serve as a link between provincial and county-level governments and play a significant role in promoting coordinated urban–rural development (49). Compared to township and county governments, prefecture-level governments have more complete administrative structures and governance capacities. Their work reports comprehensively reflect local government policy planning and implementation in various areas such as economics, society, and culture. Moreover, from an operational perspective, prefecture-level government work reports are more standardized, with more complete and detailed information, making them suitable for word frequency analysis and quantitative assessment. To facilitate interpretation of the regression coefficients, we multiply the OG by 1,000.

**Table 2:** Government aging governance terms.

Category of terms	Specific terms
General terms	Older adult, older adult people, aging, aging society, aged society, super-aged society, aging industry, general standards for the aging industry, support and assurance standards for the aging industry.
Older adult categories	Older adult people, empty nesters, solitary older adult, bereaved older adult, older adult in extreme poverty, older adult under minimum living allowance, older adult at home, self-sufficient older adult, disabled older adult, cognitively impaired older adult, assisted older adult, care-dependent older adult.
Older adult care models	Institutional older adult care, community older adult care, home-based older adult care, socialized older adult care, travel-based older adult care, inclusive older adult care, community-embedded older adult care services, smart older adult care, Internet plus older adult care, public-private older adult care, government-run, privately managed older adult care.
Older adult care facilities	Older adult care facilities, older adult care institutions, nursing homes, older adult social welfare institutes, older adult care centers, older adult asylums, rural happiness homes, home-based older adult care service facilities, community older adult care institutions, older adult care service guidance centers, community older adult service stations, travel-based older adult care institutions, nursing centers, day care centers, older adult care facilities, full-day older adult care facilities, day care facilities for older adult people, community day care centers for the older adult, older adult residential buildings, accessible pathways, older adult housing, older adult apartments, family-oriented rooms, home hospital beds, open-air nursing homes, older adult schools, older adult activity centers, comprehensive community older adult care service information platforms.
Services and products	Older adult care services, older adult care service provision standards, older adult care service systems, older adult care service standards, older adult education, older adult health education, older adult cultural and recreational activities, older adult sports activities, older adult-friendly environment renovation, home-based older adult-friendly renovations, older adult health management, older adult capability assessment, older adult volunteer services, older adult products, older adult health products, older adult nutrition products, older adult finance, older adult records, older adult health records, older adult service records.
Older adult care personnel	Older adult social workers, older adult care volunteers, older adult health massage therapists, older adult psychological counselors, older adult nutritionists, older adult care practitioners, older adult care nurses, older adult service assessors.
Older adult rights	Older adult rights, older adult social welfare, older adult social assistance, older adult social support, basic older adult insurance, basic older adult medical insurance, comprehensive liability insurance for older adult care institutions, accidental injury insurance for older adult people, long-term older adult care insurance, reverse mortgage older adult insurance, elder abuse, older adult human resources, Older adult Day.

#### 3.2.3 Mechanism variables

The mechanism variables include quality of grassroots democracy and network conditions. Quality of grassroots democracy (GD) based on indicator “Has anyone in your household participated in voting for the village committee election?”. “What is the Internet condition at your home” is used as the proxy variable for Internet condition (NC).

#### 3.2.4 Control variables

This study focuses on examining the impact of government aging governance on the multidimensional relative poverty of households. Therefore, other factors that may affect rural multidimensional relative poverty are included as control variables, encompassing household-level and prefecture-level variables. The specific variables are shown in Table 3.

**Table 3:** Variable selection and explanation.

Variable category	Level	Variable name	Symbol	Variable description
Dependent variable	Household	Multidimensional relative poverty	*MP*	1 = farm household in multidimensional relative poverty, 0 = not in
Core explanatory variables	City	Government governance of aging	*OG*	Frequency of government aging governance terminology in government work reports
Mechanism variables	Household	Quality of grassroots democracy	*GD*	1 = Someone in the family voted in the village committee election, 2 = none
		Network conditions	*NC*	1 (good) to 3 (poor)
Control variable	Household	Sex of head of household	*GEN*	1 = Male; 0 = Female
		Age of head of household	*AGE*	Age (years)
		Household size	*FAM*	Number of household members (persons)
		Older adult	*OLD*	Percentage of household members aged 65 or above (%)
		Children	*CHI*	Percentage of household members under 14 years old (%)
	City	Industrial structure	*IND*	Value added of secondary industry / value added of tertiary industry (%)
		Consumption level	*CON*	Total retail sales of consumer goods/GDP (%)

### 3.3 Data sources

The data used in this study primarily come from the China Rural Revitalization Survey (CRRS) conducted by the Institute of Rural Development at the Chinese Academy of Social Sciences, the China City Statistical Yearbook, and the government work reports from various prefecture-level cities in China. After data cleaning and processing, the government aging governance level, prefecture-level control variables, and CRRS data were matched and integrated into pool data for 10 provinces covering the years 2020 and 2022.

## 4 Empirical results and analysis

### 4.1 Baseline regression results

The baseline regression results are shown in Table 4. Columns (1) to (3) present the baseline regression results with the sequential addition of time fixed effects and city fixed effects. The results indicate that the coefficient of government aging governance is significantly negative at the 1, 10, and 10% levels, with coefficients of 0.5827, 0.1971, and 0.2131, respectively, suggesting that government aging governance significantly reduces the level of rural multidimensional relative poverty.

**Table 4:** Benchmark regression results.

Category of variables	Variables	Explained variables		
		(1)	(2)	(3)
Explanatory variables	OG	−0.5827***	−0.1971*	−0.2131*
		(0.0818)	(0.1196)	(0.1194)
Household level control variables	GEN	0.0647***	0.0564**	0.0538**
		(0.0235)	(0.0232)	(0.0231)
	AGE	0.0034***	0.0038***	0.0043***
		(0.0007)	(0.0006)	(0.0006)
	FAM	−0.0336***	−0.0326***	−0.0303***
		(0.0042)	(0.0042)	(0.0042)
	OLD	0.4215***	0.4265***	0.4215***
		(0.0469)	(0.0452)	(0.0452)
	CHI	0.2019***	0.2023***	0.2116***
		(0.0242)	(0.0236)	(0.0234)
City level control variables	IND	0.0696***	0.1651***	0.2276***
		(0.0143)	(0.0303)	(0.0329)
	CON	−0.1943***	0.2865	0.1333
		(0.0641)	(0.1768)	(0.1783)
	Constant	0.4177***	0.2999***	0.3274***
		(0.0514)	(0.0790)	(0.0782)
	City fixed effects	No	Yes	Yes
	Time fixed effects	Yes	No	Yes
	N	6,754	6,754	6,754
	adj. $R^{2}$	0.056	0.132	0.141

*p < 0.1, **p < 0.05, ***p < 0.01; the numbers in parentheses are regression standard errors.

Examining the control variables reveals the following insights: the estimated coefficient of household head’s gender (GEN) is significantly positive at the 5% level, with a coefficient of 0.0538, indicating that “female-headed” households are better at alleviating multidimensional relative poverty than “male-headed” households. The coefficient for household head’s age (AGE) is significantly positive at the 1% level, with a coefficient of 0.0043, implying that an older household head may exacerbate multidimensional relative poverty. Household size (FAM) has a significantly negative coefficient at the 1% level, with a coefficient of −0.0303, indicating that larger household size helps alleviate multidimensional relative poverty. The estimated coefficients for the proportion of older adult (OLD) and the proportion of children (CHI) are significantly positive at the 1% level, with coefficients of 0.4215 and 0.2116, respectively, showing that families with higher proportions of older adult and children are more susceptible to multidimensional relative poverty. The coefficient for industrial structure (IND) is significantly positive at the 1% level, with a coefficient of 0.2276, suggesting that rural areas with a higher proportion of secondary industry relative to tertiary industry are more likely to experience multidimensional relative poverty.

### 4.2 Endogeneity test

Omitted variables and bidirectional causality can result in endogeneity issues. To address this, this study identifies a suitable instrumental variable and applies the two-stage least squares (2SLS) method for estimation. The instrumental variable used is regional aging attention (AT), constructed from the annual average search frequencies of four keywords “aging,” “older adult,” “senior citizens,” and “old people”—on both mobile and desktop platforms, based on Baidu Search Index data. Aging attention is a valid instrumental variable for government aging governance for two reasons. First, the higher the attention a region’s residents pay to aging issues, the more importance they place on aging problems, which incentivizes the government to take more proactive measures in aging governance, satisfying the relevance assumption for instrumental variables. Second, aging attention is influenced by the region’s aging population and family caregiving burdens, which are exogenous factors that do not directly affect rural multidimensional poverty levels, satisfying the exogeneity assumption for instrumental variables. Given that the values for aging attention are relatively large, a logarithmic transformation of aging attention (LAT) is applied to stabilize the data, which is used as the instrumental variable for government aging governance.

Table 5 reports the 2SLS regression results using LAT as the instrumental variable. Column (1) shows the first-stage regression results, where the regression coefficient for the instrumental variable, L. AT, is significantly positive at the 1% level, indicating a significant positive correlation between aging attention and government aging governance, consistent with expectations. The following statistics were used to test the validity of the instrumental variable: (1) The F-statistic for the first-stage regression is 75.04, far exceeding the conventional threshold of 10, indicating no issue of weak instruments; (2) The Kleibergen-Paap rk LM statistic rejects the null hypothesis of “under-identification” at the 1% level; (3) The Kleibergen-Paap rk Wald F statistic is 75.045, which is greater than the 10% critical value of 16.38 from the Stock-Yogo weak ID test critical values, rejecting the null hypothesis of “weak identification” at a high level. These tests indicate that the instrumental variable constructed in this study is valid. Column (2) of Table 5 presents the second-stage 2SLS regression results. The estimated coefficient for multidimensional poverty (MP) remains significantly negative, suggesting that even after controlling for potential endogeneity using the instrumental variable, the suppressing effect of government aging governance on multidimensional poverty remains significant.

**Table 5:** 2SLS regression results.

Variables	(1)	(2)
	*OG*	*MP*
*L. AT*	0.0097***	
	(0.0011)	
*OG*		−6.2204***
		(0.9448)
Control variable	Yes	Yes
City fixed effects	Yes	Yes
Time fixed effects	Yes	Yes
*N*	6,752	6,752

*p < 0.1, **p < 0.05, ***p < 0.01; the numbers in parentheses are regression standard errors. Due to space constraints, the table of control variables has been omitted from this paper.

### 4.3 Robustness tests

#### 4.3.1 Replacing the key explanatory variable

In calculating the frequency and total text length for government aging governance, this paper uses the default mode of the jieba word segmentation library in Python for word segmentation. In this default mode, the jieba library performs full-word matching on the sentence to be segmented, identifying all possible words and then calculating the most likely segmentation based on the word frequency in the dictionary. To achieve more precise segmentation and statistics, this paper employs the jieba library’s “search engine mode” for text segmentation and calculates the level of government aging governance under this mode, denoted as *OGS*, as a robustness check by replacing the original measure, *OG*. The regression results are presented in Column (1) of Table 6. It can be observed that, even after replacing the core explanatory variable, the estimated coefficient of government mitigation governance on rural multidimensional poverty remains significantly negative at the 10% level. This result indicates that, with an alternative measurement for government aging governance, government efforts continue to effectively alleviate rural multidimensional poverty, supporting the robustness of the baseline regression conclusions in this paper.

**Table 6:** Robustness tests.

Variables	(1)	(2)	(3)
	*MP*	*MPPCA*	*MP* (*k*=0.4)
*OGS*	−0.0700*		
	(0.0420)		
*OG*		−0.6269***	−0.3035***
		(0.1200)	(0.1035)
Control variable	Yes	Yes	Yes
City fixed effects	Yes	Yes	Yes
Time fixed effects	Yes	Yes	Yes
*N*	6,754	6,754	6,754
adj. $R^{2}$	0.140	0.108	0.137

*p < 0.1, **p < 0.05, ***p < 0.01; the numbers in parentheses are regression standard errors. Due to space constraints, the table of control variables has been omitted from this paper.

#### 4.3.2 Changing the weighting method for the dependent variable

In this study, an equal-weighted approach is employed for measuring multidimensional relative poverty. To test the robustness of this method, principal component analysis (PCA), an objective weighting method, is applied. PCA is a statistical technique used for dimensionality reduction and feature extraction. By transforming the original high-dimensional data into a smaller set of composite variables (referred to as principal components), PCA retains as much of the data’s variance as possible. These principal components are independent of each other and are ranked according to their ability to explain the data’s variance, with the first principal component typically holding the most explanatory power. PCA aids in simplifying data structures, reducing noise, highlighting main patterns within the data, and facilitating analysis and visualization. The results of the importance of the indicators measured by the PCA method are shown in Table 7.

**Table 7:** Results of PCA weighting.

Dimension	Weight
Education level	0.0538
Health status	0.0001
Subjective feelings	0.3438
Social participation	0.6020
Income status	0.0003

Subsequently, the rural multidimensional relative poverty index, denoted as MPPCA, was calculated using the weights estimated by the PCA method. The regression results are shown in column (2) of Table 6. The results indicate that after changing the weighting method for the key dependent variable, the impact of government aging governance on rural multidimensional relative poverty remains significantly negative at the 1% level, confirming the robustness of the suppressing effect of government aging governance on rural multidimensional relative poverty.

#### 4.3.3 Changing the poverty threshold

To test whether the setting of the poverty threshold affects the conclusions of this study, the poverty threshold was changed from 0.3 to 0.4. The regression results are shown in column (3) of Table 6. The results indicate that after changing the poverty threshold, the mitigating effect of government aging governance on rural multidimensional poverty remains significantly negative at the 1% level. This suggests that changing the poverty threshold does not affect the conclusions of this study.

## 5 Heterogeneity analysis and mechanism analysis

### 5.1 Heterogeneity analysis

#### 5.1.1 Regional heterogeneity analysis

The results in columns (1) and (2) of Table 8 show significant regional heterogeneity in the effect of government aging governance on alleviating rural multidimensional relative poverty. Specifically, the estimated coefficients for the eastern and central-western regions are 0.5079 and 0.2426, respectively, both significantly negative, indicating that there are significant regional differences in the poverty-alleviating effects of government aging governance. A possible explanation is that the eastern region has a higher level of economic development and more developed infrastructure, allowing government aging governance policies to be more effective in improving the quality of life and well-being of the older adult. Additionally, the impact of government aging governance varies across regions due to differences in local socioeconomic structures and the proportion of the older adult population. In the eastern region, where the economy is more dynamic, there is stronger policy support, and the degree of aging is relatively low, government aging governance policies can more quickly and comprehensively cover the older adult population, significantly alleviating multidimensional poverty. In contrast, in the central-western regions, due to geographic and economic limitations, the implementation and coverage of government policies face greater challenges, and thus the effects of the policies are relatively limited, requiring more attention (50).

**Table 8:** Heterogeneity analysis.

Variables	(1)	(2)	(3)	(4)
	Eastern	Central-western	Young	Old
*OG*	−0.5079***	−0.2426**	−0.6635***	−0.3084**
	(0.1293)	(0.1103)	(0.0971)	(0.1539)
Control variable	Yes	Yes	Yes	Yes
City fixed effects	Yes	Yes	Yes	Yes
Time fixed effects	Yes	Yes	Yes	Yes
*N*	2061	4,693	4,934	1820
adj. *R^2^*	0.082	0.060	0.032	0.077

*p < 0.1, **p < 0.05, ***p < 0.01; the numbers in parentheses are regression standard errors. Due to space constraints, the table of control variables has been omitted from this paper.

#### 5.1.2 Age heterogeneity analysis

Government aging governance can alleviate multidimensional poverty by improving the living standards and social security of the older adult within families, and it can also reduce multidimensional poverty in younger households by increasing employment opportunities and reducing the pressure of older adult care. Specifically, government aging governance increases welfare and social security for the older adult, easing the financial burden of older adult care and improving the overall economic status of households. In this study, households where more than 25% of the members are over 60 years old are defined as older adult households, while households with less than 25% are defined as younger households. The heterogeneity of the impact of government aging governance on younger and older adult households is examined. Columns (3) and (4) of Table 8 show the regression results for the effect of government aging governance on multidimensional relative poverty in younger and older adult households, respectively. The results indicate that both are significantly negative at the 10% level, and the impact of government aging governance on the alleviation of multidimensional poverty in young and middle-aged families (0.6635) is greater than that on older adult families (0.3084). A possible explanation for this is that young and middle-aged families benefit more from these policies. Improvements in public healthcare facilities and increased government subsidies reduce the caregiving burden on young and middle-aged families, while also providing them with more employment opportunities (such as older adult care) (51), thereby alleviating multidimensional relative poverty.

### 5.2 Mechanism analysis

In order to verify H2 and H3, this paper draws on Chen, Fan (52)’s methodology and conducts mechanism testing via stepwise regression. We first regress the relationship between the core dependent variable, rural multidimensional relative poverty (MP), and the core explanatory variable, aging government governance (OG), a step we have already completed in the benchmark regression. Second, we sequentially regress the mechanism variables, grassroots democratic quality (GD) and network conditions (NC), as the dependent variables, and aging government governance (OG) as the explanatory variable on the two mechanisms. Regressions, respectively, to determine whether aging government governance can effectively contribute to the improvement of grassroots democratic quality and network conditions, thereby alleviating multidimensional rural relative poverty.

#### 5.2.1 Quality of grassroots democracy

This paper uses the indicator “Has anyone in your household participated in voting for the village committee election?” to measure the quality of grassroots democracy (GD). The results in Column (1) of Table 9 indicate that the estimated coefficient of government aging governance on the level of grassroots rural governance is significantly negative at the 1% level, with an estimated coefficient of 0.5124. This suggests that government aging governance can alleviate rural multidimensional relative poverty by enhancing the quality of grassroots democracy.

**Table 9:** Mechanism analysis.

Variables	(1)	(2)
	*GD*	*NC*
*OG*	−0.5124***	−1.0311***
	(0.1037)	(0.2070)
Control variable	Yes	Yes
City fixed effects	Yes	Yes
Time fixed effects	Yes	Yes
*N*	6,637	6,387
adj. *R*^2^	0.264	0.146

*p < 0.1, **p < 0.05, ***p < 0.01; the numbers in parentheses are regression standard errors. Due to space constraints, the table of control variables has been omitted from this paper.

#### 5.2.2 Network conditions

This paper uses the question “How are the network conditions in your household?” from the CRRS questionnaire as a proxy variable for network conditions (NC). The estimation result in Column (2) of Table 9 shows that the coefficient of government aging governance on network conditions is significantly negative at the 1% level, with an estimated coefficient of 1.0311. This indicates that government aging governance can improve network conditions, thereby alleviating rural multidimensional poverty.

## 6 Conclusion and policy recommendations

### 6.1 Conclusion

Based on data from the “China Rural Revitalization Survey” (CRRS) conducted by the Institute of Rural Development at the Chinese Academy of Social Sciences, this study examines the impact of government aging governance on rural multidimensional relative poverty. The results show that: (1) The baseline regression results indicate that government aging governance significantly reduces the level of rural multidimensional relative poverty. Through measures such as improving the pension system, strengthening social security, and enhancing infrastructure development, the quality of life and well-being of the older adult have been improved, while the burden of older adult care on households has been reduced. (2) The heterogeneity analysis reveals that households in the eastern region and households with a higher proportion of young people benefit more significantly from reduced multidimensional relative poverty. This suggests that regional characteristics and the specific needs of target groups should be considered in policy formulation and implementation to ensure effectiveness and equity. (3) Government aging governance alleviates multidimensional poverty by improving grassroots democratic quality and enhancing digital infrastructure in rural areas. The improvement of grassroots democratic quality ensures policy transparency and enforcement, while the promotion of digital infrastructure greatly facilitates rural older residents’ lives, enhancing their ability to access information and participate in social activities, thereby improving their overall quality of life and economic status.

### 6.2 Policy recommendations

Based on the study’s conclusions, the following policy recommendations are proposed: Firstly, with the complete victory in China’s poverty alleviation campaign, rural multidimensional relative poverty has become the main form of poverty among households. The government should establish a multidimensional relative poverty assessment system tailored to local conditions to accurately identify and assist poor populations. In terms of education, efforts should be made to enhance the availability and quality of educational resources in rural areas. Digital technologies such as distance education and online learning can help narrow the urban–rural education gap. In terms of health, the construction of rural healthcare infrastructure should be strengthened, along with improving basic healthcare services and the rural medical insurance system. Regarding subjective well-being, increasing the sense of happiness and satisfaction among farmers through community activities and psychological support services can improve the quality of life and mental health of households. In terms of social participation, encouraging rural households to participate in social governance and public decision-making can enhance their sense of belonging and civic consciousness. In terms of income, skills training and microcredit support can help households develop new income sources, improve income levels, and achieve economic self-sufficiency.

Secondly, improve governance systems and enhance the level of government aging governance. Currently, the government’s awareness of aging issues is still insufficient, and aging governance has not yet been effectively integrated into the national governance system. Therefore, it is urgently necessary to strengthen local governments’ scientific understanding of aging issues in the governance process and integrate aging governance strategies with law, economics, society, government, community, rural areas, public security, and global governance. Promoting the modernization of aging governance strategies and accelerating the national layout for aging governance modernization are also essential.

Thirdly, enhance grassroots democratic quality and digital infrastructure development. Through institutional innovation and management optimization, the transparency and accountability of village committees should be improved, and training for village committees and collective personnel should be strengthened to better respond to the needs of rural households. A more comprehensive feedback mechanism and regular community meetings should be established to enhance self-management capabilities in rural areas and increase residents’ awareness of participation. Additionally, rural digital infrastructure development should be strengthened. The government should promote comprehensive digital infrastructure upgrades and share the benefits of the digital economy with rural households. Stable internet access services should be provided, and smart agricultural technologies and e-commerce platforms should be promoted to facilitate broader sales of agricultural products and more efficient agricultural management. Digital tools can also be used to improve online education resources and telemedicine services to address educational and healthcare issues in rural areas.

## Data Availability

Publicly available datasets were analyzed in this study. This data can be found here: the datasets analyzed during our study are from the China Rural Revitalization Survey conducted by Rural Development Institute, Chinese Academy of Social Sciences (http://rdi.cass.cn/dcsj/202306/t20230607_5643271.shtml). However, access to the CRRS Database is subject to restrictions and requires a license. Interested researches can obtain these data with permission from the CRRS Database. Prefectural level data from China City Statistical Yearbook data (https://cnki.nbsti.net/CSYDMirror/area/Yearbook/Single/N2006020064?z=D05) and the prefectural government work report. The work reports of prefecture-level governments are open data and can be downloaded from the official websites of local governments.
